# Expression and prognosis analyses of BUB1, BUB1B and BUB3 in human sarcoma

**DOI:** 10.18632/aging.202944

**Published:** 2021-04-19

**Authors:** Zeling Long, Tong Wu, Qunyan Tian, Luke A. Carlson, Wanchun Wang, Gen Wu

**Affiliations:** 1 Department of Orthopedics, The Second Xiangya Hospital of Central South University, Changsha, Hunan, China; 2 University of Pittsburgh, Pittsburgh, PA 15260, USA

**Keywords:** budding uninhibited by benzimidazoles, sarcoma, oncogene

## Abstract

Budding Uninhibited By Benzimidazoles are a group of genes encoding proteins that play central roles in spindle checkpoint during mitosis. Improper mitosis may lead to aneuploidy which is found in many types of tumors. As a key mediator in mitosis, the dysregulated expression of BUBs has been proven to be highly associated with various malignancies, such as leukemia, gastric cancer, breast cancer, and liver cancer. However, bioinformatic analysis has not been applied to explore the role of the BUBs in sarcomas. Herein, we investigate the transcriptional and survival data of BUBs in patients with sarcomas using Oncomine, Gene Expression Profiling Interactive Analysis, Cancer Cell Line Encyclopedia, Kaplan-Meier Plotter, LinkedOmics, and the Database for Annotation, Visualization and Integrated Discovery. We found that the expression levels of BUB1, BUB1B and BUB3 were higher in sarcoma samples and cell lines than in normal controls. Survival analysis revealed that the higher expression levels of BUB1, BUB1B and BUB3 were associated with lower overall and disease-free survival in patients with sarcomas. This study implies that BUB1, BUB1B and BUB3 are potential treatment targets for patients with sarcomas and are new biomarkers for the prognosis of sarcomas.

## INTRODUCTION

Incorrect segregation of sister chromatids during mitosis may lead to aneuploidy which is found in many types of tumors [1, 2]. The Budding Uninhibited By Benzimidazoles (BUB1, BUB1B, and BUB3) gene family plays a central role in spindle checkpoint during mitosis [3–6].

BUB1 is required for chromosome congression, kinetochore localization, and the establishment and/or maintenance of efficient bipolar attachment to spindle microtubules [7, 8]. As a paralogous gene of BUB1, BUB1B associates with unattached/incorrectly attached kinetochores, stabilizes kinetochore-microtubule attachment, and helps ensure proper chromosome alignment [9, 10]. BUB3 works when binding with BUB1. When the spindle assembly checkpoint is activated, the BUB1/BUB3 complex is formed. It inhibits the ubiquitin ligase of APC/C (anaphase promoting complex C) by phosphorylation of its activator - CDC20 [11, 12].

In the process of mitosis, human cells show a protein phosphorylation peak that changes the behavior of a large group of proteins. In the meantime, process of mitosis also promotes the dramatic transformation of cell morphology, intracellular structure, and biochemistry [6]. Accurate chromosomal segregation is essential to mitosis. It ensures that chromosomes are properly aligned and attached to microtubules with their kinetochores at metaphase. Errors in this process may result in chromosomal mis-segregation and subsequent mitotic catastrophes, leading to chromosomal instability and the formation of tetraploid or aneuploid cells [13]. As a key mediator of the spindle checkpoint, deregulation of BUBs has been shown to be highly associated with various malignancies such as leukemia, gastric cancer, breast cancer, and liver cancer [6, 14–19].

Sarcoma is a malignant cancer consisting of a heterogeneous group of mesenchymal neoplasms [20]. The degradation of the mitotic checkpoint kinase BUB1 has been shown in Kaposi’s sarcoma [21]. However, bioinformatic analysis has not been applied to explore the role of the BUB family in other types of sarcomas. A comprehensive analysis of BUBs in sarcomas may provide a novel tool for the prognosis of the disease and may potentially be valuable therapeutic targets.

Through analysis of gene expression data from publicly-available datasets, we explored the expression of different BUB factors in patients with sarcoma to determine their expression patterns, distinct prognostic values, and potential functions in sarcoma.

## RESULTS

### Transcriptional levels of BUBs in patients with sarcoma

**Figure 1:**
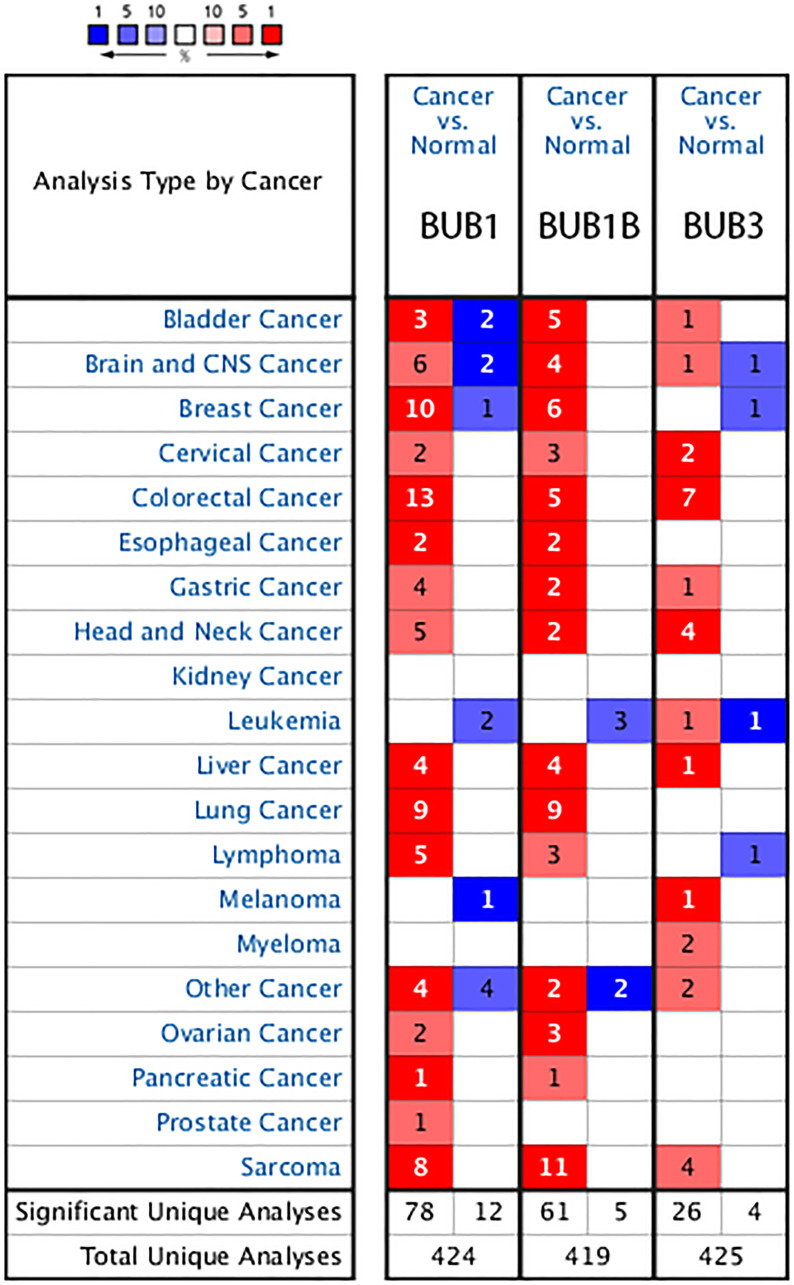
**The transcription levels of BUB factors in different types of cancers (Oncomine).** The BUB family of genes are overexpressed in many types of cancers including Sarcoma. The red cells represent evidence of gene overexpression. The blue cells represent evidence of decreased gene expression. The number in each cell represents the amount of evidence. The deeper the color, the higher the significance.

Three BUB family members have been identified in patients with different types of sarcoma: BUB1, BUB1B and BUB3. They are overexpressed in all samples of sarcoma compared to normal tissue. The transcriptional levels of BUBs were compared between sarcoma and normal samples using Oncomine databases (Figure 1). The mRNA levels of these BUBs were significantly upregulated in patients with sarcoma in two datasets.

In the Detwiller Sarcoma dataset, BUB1 was upregulated in malignant fibrous histiocytoma, fibrosarcoma, leiomyosarcoma, round cell liposarcoma, and pleomorphic liposarcoma with 12.013 (*P* = 1.59E-09), 14.408 (*P* = 2.70E-08), 11.066 (*P* = 3.42E-07), 9.248 (*P* = 2.85E-06), and 10.159 (*P* = 6.81E-05) fold changes, respectively. BUB1B was upregulated in fibrosarcoma, pleomorphic liposarcoma, malignant fibrous histiocytoma, round cell liposarcoma, synovial sarcoma, and leiomyosarcoma. The fold changes for upregulation of each sarcoma are as follows: 8.908 (*P* = 2.52E-07), 4.687 (*P* = 6.25E-06), 5.849 (*P* = 3.01E-07), 4.734 (*P* = 1.99E-06), 3.933 (*P* = 1.13E-05), and 5.471 (*P* = 2.31E-05), respectively. BUB3 was upregulated in synovial sarcoma, fibrosarcoma, and malignant fibrous histiocytoma. The fold changes are 2.999 (*P* = 1.14E-05), 2.164 (*P* = 2.64E-05), and 2.350 (*P* = 7.05E-05), respectively. In the Barretina Sarcoma dataset, BUB1 was upregulated in myxofibrosarcoma, pleomorphic liposarcoma, and leiomyosarcoma with 3.085 (*P* = 6.53E-14), 2.926 (*P* = 1.22E-09), and 2.878 (*P* = 5.95E-09) fold changes, respectively. BUB1B was upregulated in dedifferentiated liposarcoma, myxofibrosarcoma, pleomorphic liposarcoma, leiomyosarcoma, and myxoid/round cell liposarcoma with fold changes of 3.628 (*P* = 2.20E-17), 7.544 (*P* = 1.11E-17), 5.121 (*P* = 8.66E-13), 4.838 (*P* = 1.46E-10), and 2.842 (*P* = 1.88E-10), respectively. BUB3 was upregulated in myxofibrosarcoma with a 2.514 (*P* = 1.57E-12) fold change (Table 1).

**Table 1:** The significant changes of BUBs expression in transcription level between different types of sarcomas (ONCOMINE database).

**Gene ID**	**Types of Sarcoma versus Normal**	**Fold Change**	***p*-Value**	***t*-Test**	**References**
**BUB1**	Malignant Fibrous Histiocytoma vs. Normal	12.013	1.59E–09	9.949	**Detwiller Sarcoma**
	Fibrosarcoma vs. Normal	14.408	2.70E–08	10.002	**Detwiller Sarcoma**
	Leiomyosarcoma vs. Normal	11.066	3.42E–07	9.000	**Detwiller Sarcoma**
	Round Cell Liposarcoma vs. Normal	9.248	2.85E–06	8.909	**Detwiller Sarcoma**
	Pleomorphic Liposarcoma vs. Normal	10.159	6.81E–05	8.788	**Detwiller Sarcoma**
	Myxofibrosarcoma vs. Normal	3.085	6.53E–14	11.255	**Barretina Sarcoma**
	Pleomorphic Liposarcoma vs. Normal	2.926	1.22E–09	8.750	**Barretina Sarcoma**
	Leiomyosarcoma vs. Normal	2.878	5.95E–09	7.802	**Barretina Sarcoma**
**BUB1B**	Dedifferentiated Liposarcoma vs. Normal	3.628	2.20E–17	12.579	**Barretina Sarcoma**
	Myxofibrosarcoma vs. Normal	7.544	1.11E–17	16.533	**Barretina Sarcoma**
	Pleomorphic Liposarcoma vs. Normal	5.121	8.66E–13	13.250	**Barretina Sarcoma**
	Leiomyosarcoma vs. Normal	4.838	1.46E–10	9.806	**Barretina Sarcoma**
	Myxoid/Round Cell Liposarcoma vs. Normal	2.842	1.88E–10	10.778	**Barretina Sarcoma**
	Fibrosarcoma vs. Normal	8.908	2.52E–07	7.540	**Detwiller Sarcoma**
	Pleomorphic Liposarcoma vs. Normal	4.687	6.25E–06	6.348	**Detwiller Sarcoma**
	Malignant Fibrous Histiocytoma vs. Normal	5.849	3.01E–07	7.079	**Detwiller Sarcoma**
	Round Cell Liposarcoma vs. Normal	4.734	1.99E–06	6.976	**Detwiller Sarcoma**
	Synovial Sarcoma vs. Normal	3.933	1.13E–05	5.772	**Detwiller Sarcoma**
	Leiomyosarcoma vs. Normal	5.471	2.31E–05	5.623	**Detwiller Sarcoma**
**BUB3**	Myxofibrosarcoma vs. Normal	2.514	1.57E–12	10.808	**Barretina Sarcoma**
	Synovial Sarcoma vs. Normal	2.999	1.14E–05	5.854	**Detwiller Sarcoma**
	Fibrosarcoma vs. Normal	2.164	2.64E–05	5.391	**Detwiller Sarcoma**
	Malignant Fibrous Histiocytoma vs. Normal	2.350	7.05E–05	4.687	**Detwiller Sarcoma**

**Figure 2:**
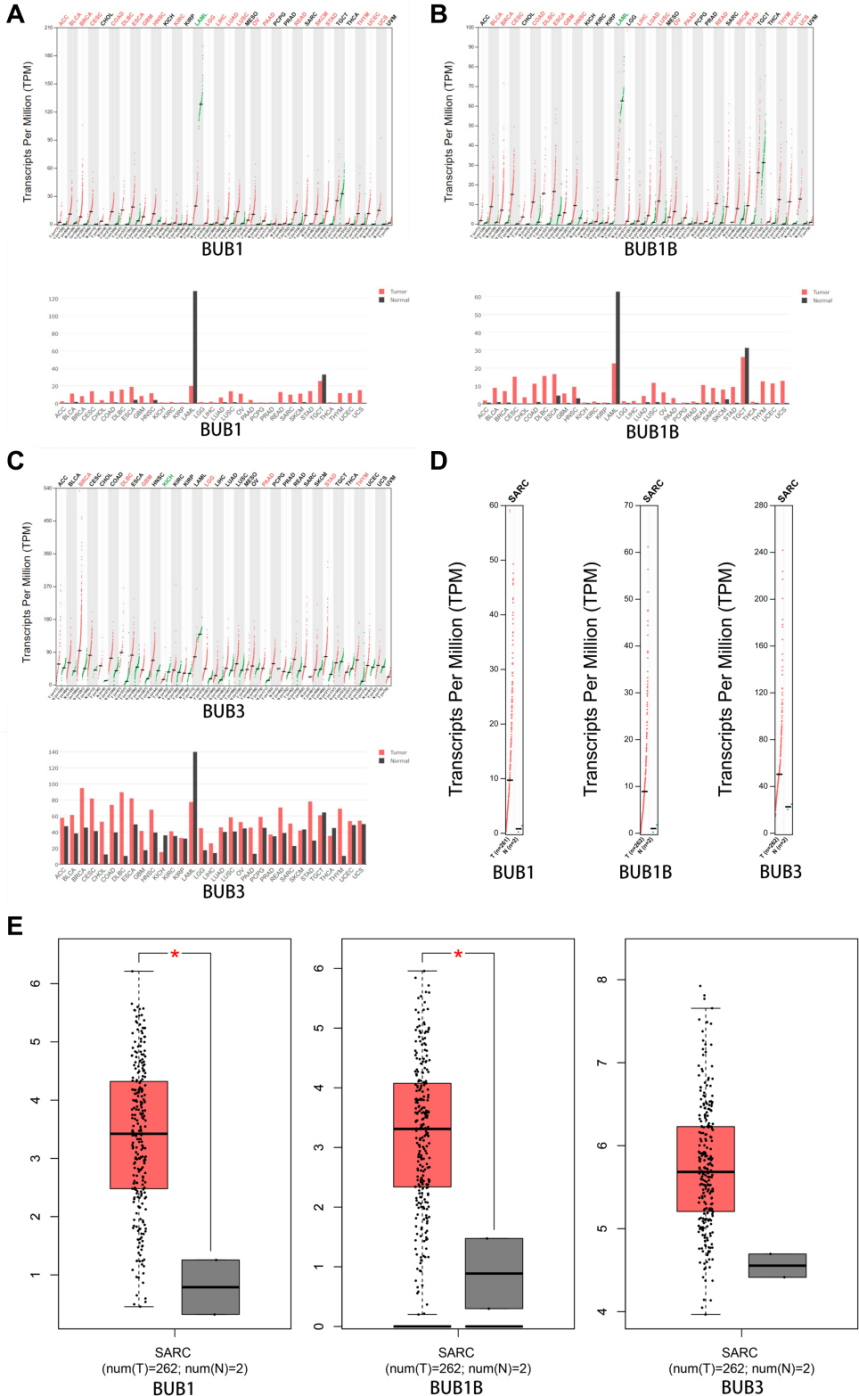
**The expression of BUBs in different types of cancers (GEPIA).** The expression of (**A**) BUB1, (**B**) BUB1B, and (**C**) BUB3 in different types of cancers. (**D**, **E**) The expression of BUBs in sarcomas. Each dot represents a sample. Cancer names were abbreviated. Details were listed in Supplementary Materials. ^*^P < 0.05.

The expression patterns of BUBs in sarcoma samples were further compared to normal tissue using the GEPIA database. Notably, the expressions of BUB1 and BUB1B are significantly higher in sarcomas compared to normal tissues (Figure 2A–2E). The overexpression of BUB3 does not reach significance, possibly due to the low number of patients in the control group.

### Transcriptional level of BUBs in sarcoma cell lines

**Figure 3:**
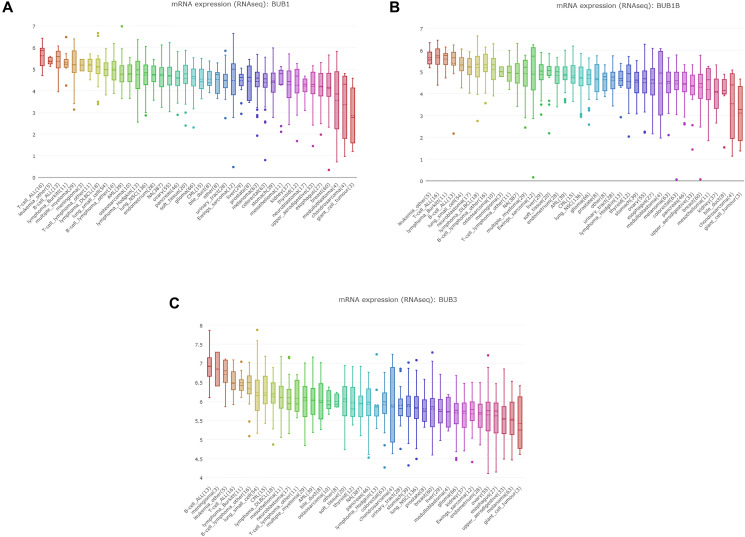
**The expression of BUBs in cancer cell lines (CCLE).** The mRNA expression of (**A**) BUB1, (**B**) BUB1B, and (**C**) BUB3 in sarcoma cell lines, analyzed by CCLE. Lineages are composed of a number of cell lines from the same area or system of the body. The number next to the lineage name indicates how many cell lines are in the lineage. The highest average distribution is on the left and is colored red. The dashed line within a box is the mean.

To further explore the expression level of BUBs in sarcoma cell lines, we queried the CCLE database. The expressions of BUB1, BUB1B and BUB3 were highly upregulated (Figure 3) compared to normal control cell lines.

### The prognostic values of BUBs in sarcoma

**Figure 4:**
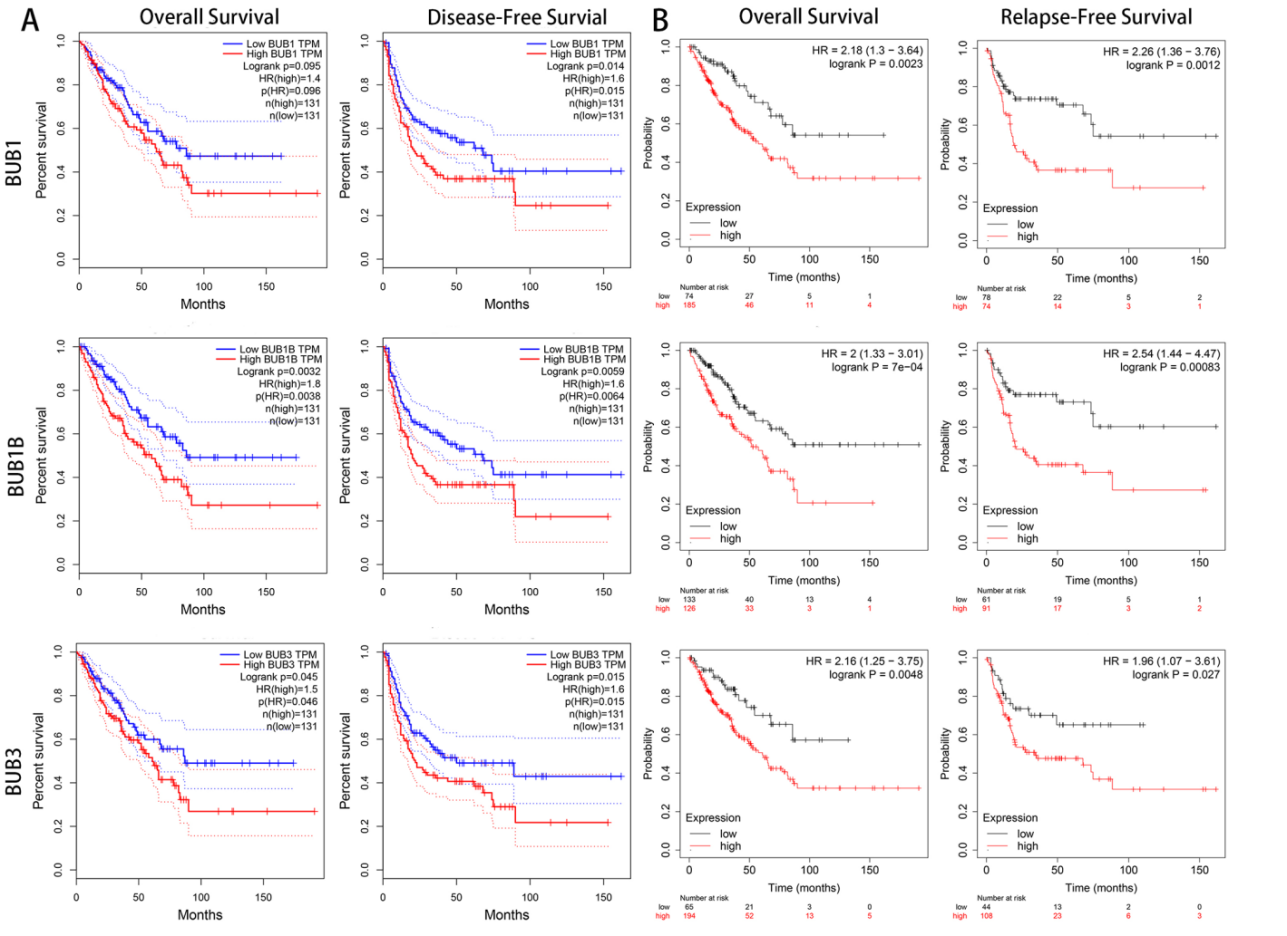
**The prognostic value of mRNA level of BUB factors in sarcoma patients (GEPIA and kaplan meier plotter).** The prognostic value of mRNA level of BUB factors in sarcoma patients was analyzed by (**A**) GEPIA and (**B**) Kaplan Meier Plotter. Higher expression of BUBs is associated with worse survival. HR, hazard ratio; TPM, Transaction per million.

To investigate the clinical prognostic value of BUBs in sarcoma, we analyzed survival data of sarcoma patients using Kaplan-Meier Plotter and the GEPIA database (Figure 4). Both overall survival and disease-free survival of sarcoma patients are negatively associated with BUBs in the datasets. In the GEPIA dataset, the hazard ratios of BUB1 are 1.4 (*P* = 0.096) for overall survival and 1.6 (*P* = 0.015) for disease-free survival. As for BUB1B, they are 1.8 (*P* = 0.0038) and 1.6 (*P* = 0.0064) for overall and disease-free survivals, respectively. For BUB3, they are 1.5 (*P* = 0.046) and 1.6 (*P* = 0.015) for overall and disease-free survivals, respectively. In the Kaplan-Meier Plotter dataset, the hazard ratios of BUB1 are 2.18 (*P* = 0.0023) for overall survival and 2.26 (*P* = 0.0012) for disease-free survival. As for BUB1B, they are 2 (*P* = 7e-04) and 2.54 (*P* = 0.00083) for overall and disease-free survivals, respectively. For BUB3, they are 2.16 (*P* = 0.0048) and 1.96 (*P* = 0.027) for overall and disease-free survivals, respectively.

### Co-expression profile of BUBs in sarcoma

**Figure 5:**
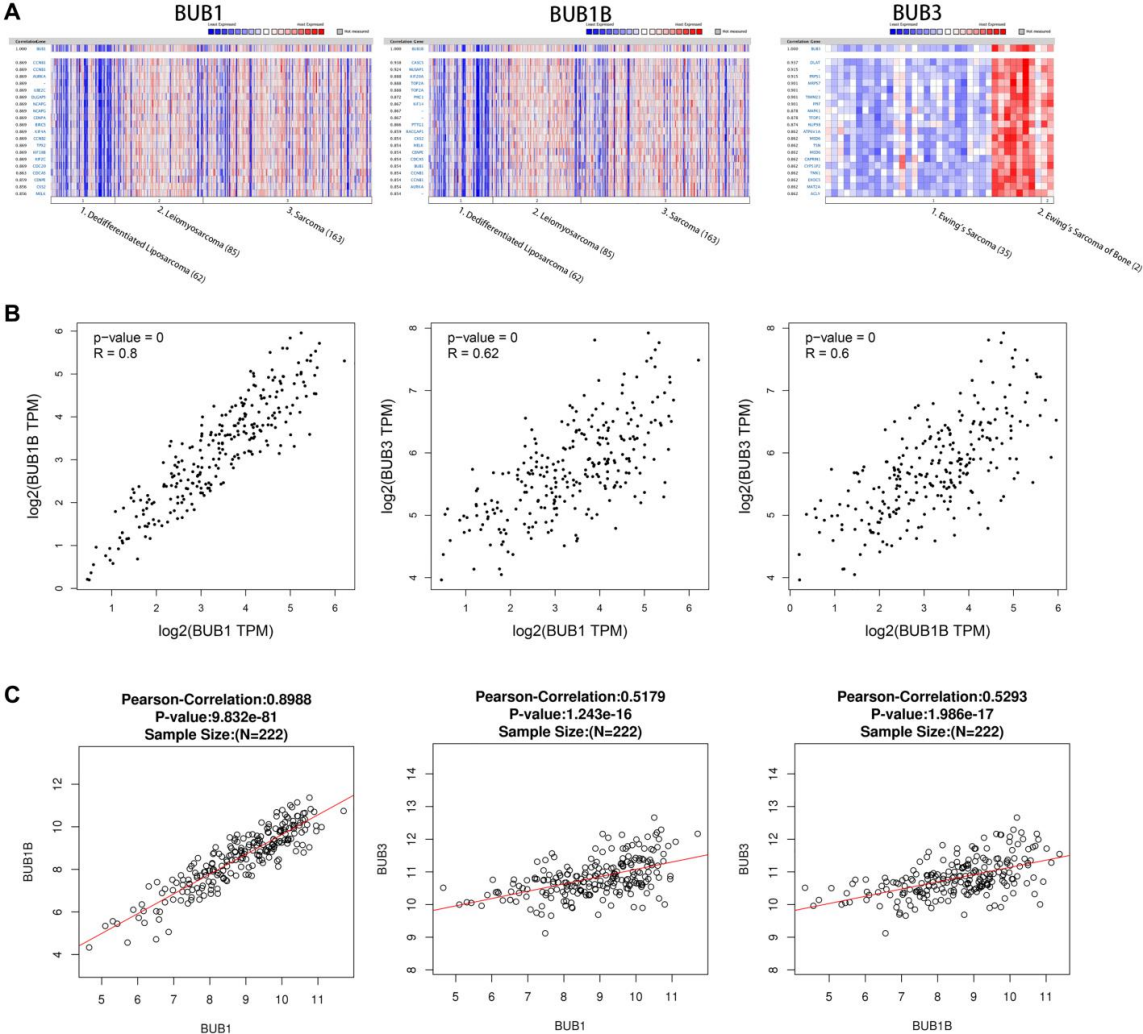
**Co-expressed genes of BUBs, correlations between BUBs (ONCOMINE, GEPIA, and LinkedOmics).** Co-expressed genes of BUBs in sarcomas, analyzed by (**A**) ONCOMINE. For each BUB factor, the top twenty co-expressed genes were displayed. The correlations between BUBs in sarcomas were analyzed by (**B**) GEPIA and (**C**) LinkedOmics. BUB1, BUB1B and BUB3 are positively correlated with each other. TPM, Transaction per million.

Genes co-expressed with BUBs in sarcoma were analyzed using the Oncomine database. In the Chibon Sarcoma datasets [22], we found BUB1 expression level was positively correlated with levels of CCNB1, AURKA, UBE2C, DLGAP5, NCAPG, CENPA, BIRC5, KIF4A, CCNB2, TPX2, KIF18B, KIF2C, CDC20, CDCA5, CENPE, CKS2, and MELK. For BUB1B, its expression level was positively correlated with levels of CACS5, NUSAP1, KIF20A, TOP2A, PRC1, KIF14, PTTG1, RACGAP1, CKS2, MELK, CENPE, CDCA5, BUB1, CCNB1, and AURKA. In the Schaefer Sarcoma dataset, we found that BUB3 expression level was positively correlated with levels of DLAT, PRPS1, MRPS7, TIMM23, PPIF, MAPK1, TFDP1, NUP98, ATP6V1A, MED6, CAPRIN1, CYP51P2, TMX1, EXOC5, MAT2A, and ACLY (Figure 5A). The association between BUB1, BUB1B, and BUB3 was analyzed using the GEPIA (Figure 5B) and Linked Omics databases (Figure 5C). The correlation coefficients of BUB1 and BUB1B were 0.8 (GEPIA) and 0.8988 (Linked Omics). For BUB1 and BUB3, they were 0.62 (GEPIA) and 0.5179 (Linked Omics). For BUB1B and BUB3, they were 0.6 (GEPIA) and 0.5293 (Linked Omics). All correlations were found to be statistically significant.

### Predicted functions and pathways of BUBs in sarcomas

**Figure 6:**
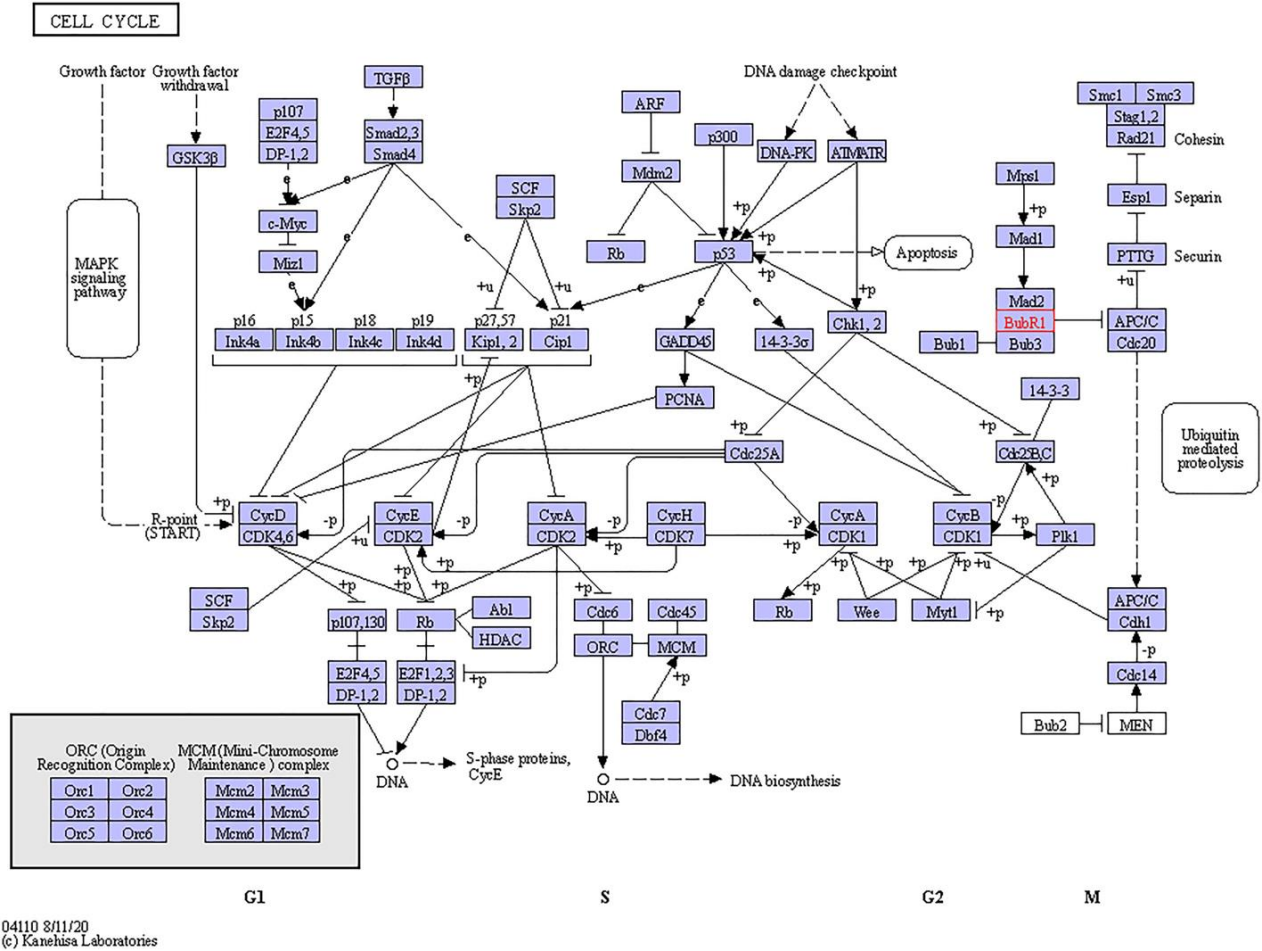
**Cell cycle regulated by the BUBs alteration in sarcomas.** The cell cycle regulated by the BUBs alteration in sarcomas are shown. Figure was downloaded from the KEGG database.

The functional roles of BUBs were evaluated from three perspectives: biological processes, molecular functions, and cellular components. We found that BUBs are involved in the biological processes of meiotic sister chromatid cohesion, meiotic cell cycle, cell division, cellular protein metabolic process, anaphase-promoting complex-dependent catabolic process, cell population proliferation, mitotic spindle assembly checkpoint, apoptotic process, protein phosphorylation, sister chromatid segregation, and nuclear division. Regarding molecular function, protein serine/threonine kinase activity was found to be significantly controlled by BUBs. Cellular components including protein-containing complex, nuclear lumen, cytosol, condensed, chromosome outer kinetochore, condensed nuclear chromosome kinetochore were all found to be associated with the function of BUBs. KEGG pathways analysis showed that BUBs are involved in Human T-lymphotropic virus 1 infection and cell cycle pathways (Table 2 and Figure 6).

**Table 2:** The functions of BUBs and genes significantly associated with BUBs alterations.

**Category**	**Term**	**Name**	**Count**	**FDR**
BP	GO:0051754	meiotic sister chromatid cohesion, centromeric	2	1.42e–06
BP	GO:0051321	meiotic cell cycle	3	4.97e–06
BP	GO:0051301	cell division	3	4.22e–05
BP	GO:0044267	cellular protein metabolic process	3	0.0107
BP	GO:0031145	anaphase-promoting complex-dependent catabolic process	2	3.01e–05
BP	GO:0008283	cell population proliferation	2	0.0062
BP	GO:0007094	mitotic spindle assembly checkpoint	3	2.16e–07
BP	GO:0006915	apoptotic process	2	0.0107
BP	GO:0006468	protein phosphorylation	2	0.0108
BP	GO:0000819	sister chromatid segregation	3	1.51e–06
BP	GO:0000280	nuclear division	3	8.96e–06
MF	GO:0004674	protein serine/threonine kinase activity	2	0.0429
CC	GO:0032991	protein-containing complex	3	0.0386
CC	GO:0031981	nuclear lumen	3	0.0277
CC	GO:0005829	cytosol	3	0.0402
CC	GO:0000940	condensed chromosome outer kinetochore	2	1.03e–05
CC	GO:0000778	condensed nuclear chromosome kinetochore	2	1.03e–05
CC	GO:0000777	condensed chromosome kinetochore	3	6.68e–06
KEGG Pathways	hsa05166	HTLV-I infection	2	0.00098
KEGG Pathways	hsa04110	Cell cycle	3	1.04e–06

The functions of BUBs and the genes significantly associated with BUBs alterations were predicted by analyzing Gene Ontology (GO) and the Kyoto Encyclopedia of Genes and Genomes (KEGG) using Database for Annotation, Visualization and Integrated Discovery (DAVID). Biological Processes (BP), Molecular Functions (MF), Cellular Components (CC).

## DISCUSSION

Dysregulation in BUB genes have been reported in many cancers [6, 14–19, 21, 23, 24]. However, the role that BUB factors play in sarcoma remains poorly understood. The present study was the first to explore the expression patterns of BUBs in sarcoma, while investigating its prognostic value and clinical relevance.

BUB1 and BUB1B are two paralogous genes that play important roles in mitosis. The catalytic function of BUB1 is required for chromosome arm resolution and positioning of the chromosomal passenger complex. BUB1B plays a role in the inhibition of the APC/C, delaying the onset of anaphase and ensuring proper chromosome segregation. Deviations from their normal expression level may cause chromosome mis-segregation, aneuploidy, and cancer predisposition. Mouse embryonic fibroblasts lacking BUB1 catalytic activity showed reduced phosphorylation of centromeric substrates of AURKB, which participate in the regulation of alignment and segregation of chromosomes during mitosis and meiosis through association with microtubules [25]. Delocalization of AURKB strongly compromises the cells' ability to efficiently correct spindle attachment errors which results in an increased rate of chromosome alignment defects [26].

The roles of BUB1/BUB1B in cancer cells are still controversial with contradicting results from studies [8]. Low expression of these genes has been shown to be associated with poor survival and cancer metastasis [27, 28], while other studies have demonstrated correlation to delayed tumor growth with prolonged survival [29, 30]. On the other hand, studies have shown that overexpression of BUB1/BUB1B is related to progression and recurrence of various tumors including glioblastoma [29], pancreatic ductal adenocarcinoma [31–33], prostate cancer [34], gastric adenocarcinoma [35], and hepatocellular carcinoma [36]. In the present study, analysis using the Oncomine and GEPIA datasets revealed that the expression of BUB1 and BUB1B were higher in human sarcomas than in normal tissues. CCLE databases also showed that BUBs were highly expressed in human sarcoma cell lines. In addition, using the Kaplan-Meier Plotter and GEPIA databases, we determined that the overall and disease-free survival of sarcoma patients were significantly lower in patients with overexpressed BUB1 and BUB1B.

BUB3 is another member of the BUB family which works by binding to BUB1. Positive correlations were found among BUB1, BUB1B, and BUB3, which is in accordance with the fact that BUBs work as a complex during mitosis. The BUB1/BUB3 complex inhibits the action of the APC, preventing early entry of anaphase and exit of mitosis, which is crucial for the fidelity of chromosomal segregation (Figure 6). This effect was produced by phosphorylation of its activator CDC20 [37]. Overexpression of BUB3 was detected in oral cancer [38], gastric cancer [14], prostate cancer [39], and lung cancer [40]. Inhibition of BUB3 could enhance tumor chemosensitivity [38]. However, in a study by Elin Ersvær et al, it was demonstrated that decreased levels of nuclear BUB3 increased the risk of tumor recurrence [39], and da Silva et al. proposed that BUB3 could exert the role of a tumor suppressor through a kinetochore-independent mechanism [41].

Despite the uncertain roles of BUB3 in cancers seen in previous studies, our study shows a positive correlation between BUB3 and human sarcomas, as well as sarcoma cell lines, especially in osteosarcoma, chondrosarcoma and Ewing sarcoma. Furthermore, higher expression of BUB3 is associated with decreased disease-free survival and overall survival.

In the present study, we systematically analyzed the expression and prognostic value of BUBs in sarcoma. Our results indicated that BUB1, BUB1B and BUB3 may serve as oncogenes in sarcomas and have significant prognostic values. Specifically, the investigation of these genes could help ensure the diagnosis of sarcomas and predict the chance of survival in patients. Highly expressed BUBs may indicate higher malignancy in sarcomas. For these patients, more radical treatment is recommended. A disadvantage of the study is that analyses were solely based on sarcoma tissue samples but not blood samples from patients. It is unknown whether BUBs are overexpressed and can be detected in the blood of sarcoma patients or not. Further exploration could be focused on the expression level of BUBs in blood samples, which may become a more convenient screening method for the diagnosis of sarcoma. In the meantime, gene therapy targeting the BUB family can be a promising approach in the treatment of sarcomas.

## MATERIALS AND METHODS

### Ethics statement

The current study was approved by the Academic Committee of Central South University and was conducted in accordance with the principles of the Declaration of Helsinki. All data sets are retrieved from published literature, all written informed consents have been obtained.

### Data collection and processing

**Figure 7:**
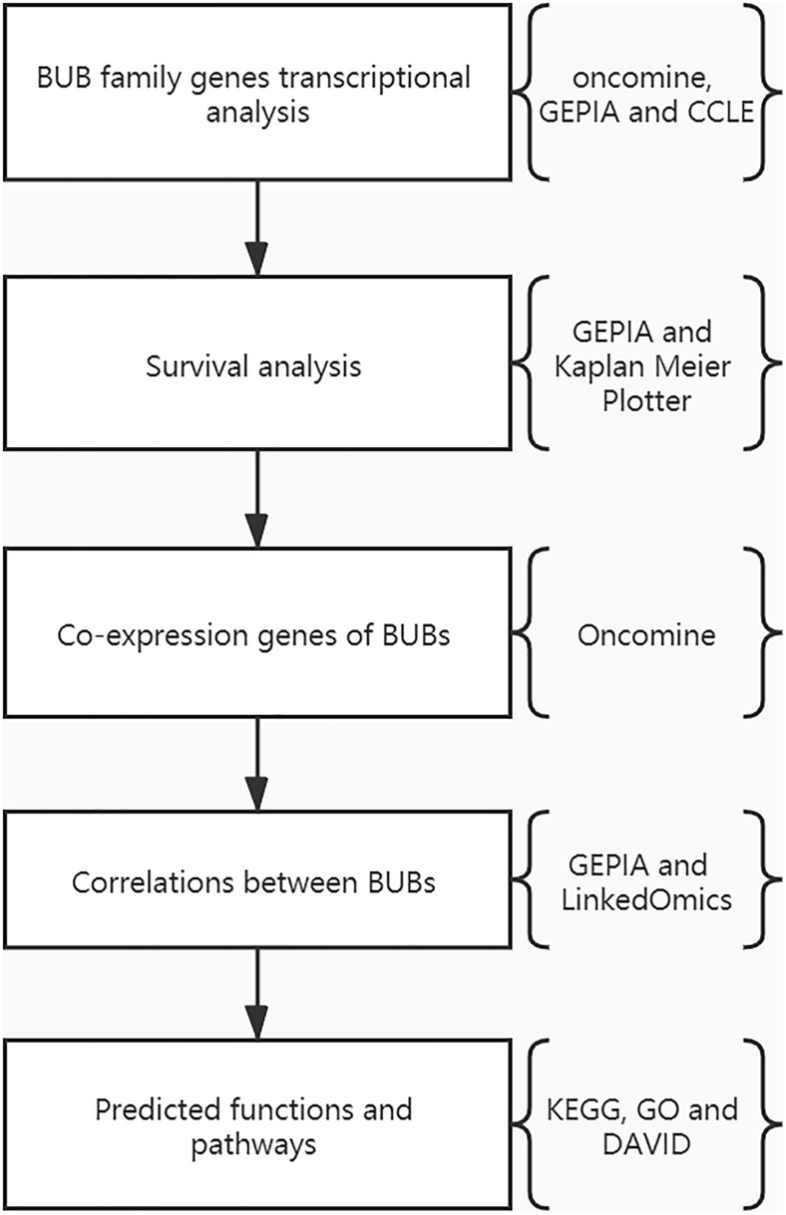
The flowchart of this study.

We first researched the expression level of the BUB gene family in tumors, especially sarcoma and normal tissues using the Oncomine database (https://www.oncomine.org/), Gene Expression Profiling Interactive Analysis (GEPIA, http://gepia.cancer-pku.cn/) and Cancer Cell Line Encyclopedia (CCLE, https://www.broadinstitute.org/ccle). Then, the prognostic value of BUBs was analyzed using GEPIA and Kaplan-Meier Plotter (https://kmplot.com/analysis/). Next, we analyzed the co-expression of BUBs in Oncomine and the correlations among BUBs in GEPIA and LinkedOmics (http://www.linkedomics.org/login.php). Lastly, the predicted functions and pathways of BUBs were analyzed in the Kyoto Encyclopedia of Genes and Genomes (KEGG, https://www.genome.jp/kegg/), Gene Ontology (GO, http://geneontology.org/), and Database for Annotation, Visualization and Integrated Discovery (DAVID, http://david.ncifcrf.gov) (Figure 7).

### ONCOMINE analysis

The transcription levels of the BUB family in different sarcomas were analyzed using Oncomine gene expression array datasets (https://www.oncomine.org/), an online cancer microarray database. Student’s *t* test was conducted to compare the mRNA expressions of the BUB family in clinical cancer specimens and normal controls [42]. The cutoff of fold change was defined as > 2. Statistical significance was set at *P* value < 0.01. Co-expressed genes of BUBs in sarcoma were analyzed using ONCOMINE.

### GEPIA dataset

Gene Expression Profiling Interactive Analysis (GEPIA, http://gepia.cancer-pku.cn/) is an interactive web platform that uses standard processing procedures to analyze the RNA sequencing expression data of 9736 tumors and 8587 normal samples from the Cancer Genome Atlas and Genotype Tissue Expression Project [43]. The expression levels of BUB family members were compared between tumor tissues and normal tissues. *P* < 0.05 was considered statistically significant.

### CCLE dataset

The Cancer Cell Line Encyclopedia (CCLE, https://www.broadinstitute.org/ccle) project is a collaboration between the Broad Institute and the Novartis Institute of Biomedicine and the Institute of Genomics of the Novartis Research Foundation to carry out detailed genetics and pharmacologic characterization on a large group of human cancer models, to develop integrated computational analysis linking different pharmacological weaknesses with genomic patterns, and to transform cell line genomics into cancer patient stratification. CCLE provides public access to genomic data, analysis and visualization for approximately 1,000 cell lines [44]. The BUB family’s expressions in cancer cell lines were confirmed by the CCLE database.

### Kaplan-Meier plotter

Kaplan Meier Plotter (https://kmplot.com/analysis/) contained the expression data of 54000 genes and the survival information of 21 cancer types including sarcoma. In order to analyze the overall survival and relapse-free survival of patients with sarcoma, the samples were divided into two groups according to median expression (BUBs high and low expression) and assessed by the Kaplan-Meier survival chart, based on hazard ratios (HR) and log-rank *P*-value [45].

### KEGG and GO enrichment analyses of DEGs

The Database for Annotation, Visualization and Integrated Discovery (DAVID, http://david.ncifcrf.gov) (version 6.7) is an online biological information database that provides a comprehensive set of functional annotation information of genes and proteins [46]. The Kyoto Encyclopedia of Genes and Genomes (KEGG, https://www.genome.jp/kegg/) is a database resource which contains large-scale molecular datasets of high-level functions and biological systems generated by high-throughput experimental technologies [47]. Gene ontology (GO) (http://geneontology.org/) is a bioinformatics tool which can annotate genes and analyze their biological processes [48]. To further analyze the function of BUBs, pathway analyses were also performed using KEGG online database.

## Supplementary Materials

Supplementary Materials

## References

[1] Mitelman F. Catalogue of chromosome aberrations in cancer. Cytogenet Cell Genet. 1983; 36:1–515. 10.1159/000131930 6627995

[2] Santaguida S, Amon A. Short- and long-term effects of chromosome mis-segregation and aneuploidy. Nat Rev Mol Cell Biol. 2015; 16:473–85. 10.1038/nrm4025 26204159

[3] Klebig C, Korinth D, Meraldi P. Bub1 regulates chromosome segregation in a kinetochore-independent manner. J Cell Biol. 2009; 185:841–58. 10.1083/jcb.200902128 19487456 PMC2711590

[4] Kiyomitsu T, Obuse C, Yanagida M. Human Blinkin/AF15q14 is required for chromosome alignment and the mitotic checkpoint through direct interaction with Bub1 and BubR1. Dev Cell. 2007; 13:663–76. 10.1016/j.devcel.2007.09.005 17981135

[5] Qi W, Yu H. KEN-box-dependent degradation of the Bub1 spindle checkpoint kinase by the anaphase-promoting complex/cyclosome. J Biol Chem. 2007; 282:3672–79. 10.1074/jbc.M609376200 17158872

[6] Zhu LJ, Pan Y, Chen XY, Hou PF. BUB1 promotes proliferation of liver cancer cells by activating SMAD2 phosphorylation. Oncol Lett. 2020; 19:3506–12. 10.3892/ol.2020.11445 32269624 PMC7114935

[7] Johnson VL, Scott MI, Holt SV, Hussein D, Taylor SS. Bub1 is required for kinetochore localization of BubR1, Cenp-E, Cenp-F and Mad2, and chromosome congression. J Cell Sci. 2004; 117:1577–89. 10.1242/jcs.01006 15020684

[8] Meraldi P, Sorger PK. A dual role for Bub1 in the spindle checkpoint and chromosome congression. EMBO J. 2005; 24:1621–33. 10.1038/sj.emboj.7600641 15933723 PMC1142573

[9] Warren CD, Brady DM, Johnston RC, Hanna JS, Hardwick KG, Spencer FA. Distinct chromosome segregation roles for spindle checkpoint proteins. Mol Biol Cell. 2002; 13:3029–41. 10.1091/mbc.e02-04-0203 12221113 PMC124140

[10] Bernard P, Hardwick K, Javerzat JP. Fission yeast bub1 is a mitotic centromere protein essential for the spindle checkpoint and the preservation of correct ploidy through mitosis. J Cell Biol. 1998; 143:1775–87. 10.1083/jcb.143.7.1775 9864354 PMC2175213

[11] Jiang H, He X, Wang S, Jia J, Wan Y, Wang Y, Zeng R, Yates J 3rd, Zhu X, Zheng Y. A microtubule-associated zinc finger protein, BuGZ, regulates mitotic chromosome alignment by ensuring Bub3 stability and kinetochore targeting. Dev Cell. 2014; 28:268–81. 10.1016/j.devcel.2013.12.013 24462186 PMC3927447

[12] Toledo CM, Herman JA, Olsen JB, Ding Y, Corrin P, Girard EJ, Olson JM, Emili A, DeLuca JG, Paddison PJ. BuGZ is required for Bub3 stability, Bub1 kinetochore function, and chromosome alignment. Dev Cell. 2014; 28:282–94. 10.1016/j.devcel.2013.12.014 24462187 PMC3995079

[13] Wang X, Cheung HW, Chun AC, Jin DY, Wong YC. Mitotic checkpoint defects in human cancers and their implications to chemotherapy. Front Biosci. 2008; 13:2103–14. 17981695 10.2741/2827

[14] Grabsch H, Takeno S, Parsons WJ, Pomjanski N, Boecking A, Gabbert HE, Mueller W. Overexpression of the mitotic checkpoint genes BUB1, BUBR1, and BUB3 in gastric cancer--association with tumour cell proliferation. J Pathol. 2003; 200:16–22. 10.1002/path.1324 12692836

[15] Hernando E, Orlow I, Liberal V, Nohales G, Benezra R, Cordon-Cardo C. Molecular analyses of the mitotic checkpoint components hsMAD2, hBUB1 and hBUB3 in human cancer. Int J Cancer. 2001; 95:223–27. 10.1002/1097-0215(20010720)95:4<223::aid-ijc1038>3.0.co;2-l 11400114

[16] Grabsch HI, Askham JM, Morrison EE, Pomjanski N, Lickvers K, Parsons WJ, Boecking A, Gabbert HE, Mueller W. Expression of BUB1 protein in gastric cancer correlates with the histological subtype, but not with DNA ploidy or microsatellite instability. J Pathol. 2004; 202:208–14. 10.1002/path.1499 14743503

[17] Mukherjee A, Joseph C, Craze M, Chrysanthou E, Ellis IO. The role of BUB and CDC proteins in low-grade breast cancers. Lancet. 2015 Suppl 1; 385:S72. 10.1016/S0140-6736(15)60387-7 26312894

[18] Ru HY, Chen RL, Lu WC, Chen JH. hBUB1 defects in leukemia and lymphoma cells. Oncogene. 2002; 21:4673–79. 10.1038/sj.onc.1205585 12096343

[19] Ohshima K, Haraoka S, Yoshioka S, Hamasaki M, Fujiki T, Suzumiya J, Kawasaki C, Kanda M, Kikuchi M. Mutation analysis of mitotic checkpoint genes (hBUB1 and hBUBR1) and microsatellite instability in adult T-cell leukemia/lymphoma. Cancer Lett. 2000; 158:141–50. 10.1016/s0304-3835(00)00512-7 10960763

[20] Skubitz KM, D'Adamo DR. Sarcoma. Mayo Clin Proc. 2007; 82:1409–32. 10.4065/82.11.1409 17976362

[21] Sun Z, Xiao B, Jha HC, Lu J, Banerjee S, Robertson ES. Kaposi's sarcoma-associated herpesvirus-encoded LANA can induce chromosomal instability through targeted degradation of the mitotic checkpoint kinase Bub1. J Virol. 2014; 88:7367–78. 10.1128/JVI.00554-14 24741095 PMC4054434

[22] Chibon F, Lagarde P, Salas S, Perot G, Brouste V, Tirode F, Lucchesi C, de Reynies A, Kauffmann A, Bui B, Terrier P, Bonvalot S, Le Cesne A, et al. Validated prediction of clinical outcome in sarcomas and multiple types of cancer on the basis of a gene expression signature related to genome complexity. Nat Med. 2010; 16:781–87. 10.1038/nm.2174 20581836

[23] Qiu J, Zhang S, Wang P, Wang H, Sha B, Peng H, Ju Z, Rao J, Lu L. BUB1B promotes hepatocellular carcinoma progression via activation of the mTORC1 signaling pathway. Cancer Med. 2020; 9:8159–72. 10.1002/cam4.3411 32977361 PMC7643650

[24] Morales AG, Pezuk JA, Brassesco MS, de Oliveira JC, de Paula Queiroz RG, Machado HR, Carlotti CG Jr, Neder L, de Oliveira HF, Scrideli CA, Tone LG. BUB1 and BUBR1 inhibition decreases proliferation and colony formation, and enhances radiation sensitivity in pediatric glioblastoma cells. Childs Nerv Syst. 2013; 29:2241–48. 10.1007/s00381-013-2175-8 23728478

[25] Ricke RM, Jeganathan KB, Malureanu L, Harrison AM, van Deursen JM. Bub1 kinase activity drives error correction and mitotic checkpoint control but not tumor suppression. J Cell Biol. 2012; 199:931–49. 10.1083/jcb.201205115 23209306 PMC3518220

[26] Baron AP, von Schubert C, Cubizolles F, Siemeister G, Hitchcock M, Mengel A, Schröder J, Fernández-Montalván A, von Nussbaum F, Mumberg D, Nigg EA. Probing the catalytic functions of Bub1 kinase using the small molecule inhibitors BAY-320 and BAY-524. Elife. 2016; 5:e12187. 10.7554/eLife.12187 26885717 PMC4769170

[27] Shichiri M, Yoshinaga K, Hisatomi H, Sugihara K, Hirata Y. Genetic and epigenetic inactivation of mitotic checkpoint genes hBUB1 and hBUBR1 and their relationship to survival. Cancer Res. 2002; 62:13–17. 11782350

[28] Park HY, Jeon YK, Shin HJ, Kim IJ, Kang HC, Jeong SJ, Chung DH, Lee CW. Differential promoter methylation may be a key molecular mechanism in regulating BubR1 expression in cancer cells. Exp Mol Med. 2007; 39:195–204. 10.1038/emm.2007.22 17464181

[29] Yu H, Zhang S, Ibrahim AN, Deng Z, Wang M. Serine/threonine kinase BUB1 promotes proliferation and radio-resistance in glioblastoma. Pathol Res Pract. 2019; 215:152508. 10.1016/j.prp.2019.152508 31272759

[30] Siemeister G, Mengel A, Fernández-Montalván AE, Bone W, Schröder J, Zitzmann-Kolbe S, Briem H, Prechtl S, Holton SJ, Mönning U, von Ahsen O, Johanssen S, Cleve A, et al. Inhibition of BUB1 Kinase by BAY 1816032 Sensitizes Tumor Cells toward Taxanes, ATR, and PARP Inhibitors *In Vitro* and *In Vivo*. Clin Cancer Res. 2019; 25:1404–14. 10.1158/1078-0432.CCR-18-0628 30429199

[31] Piao J, Zhu L, Sun J, Li N, Dong B, Yang Y, Chen L. High expression of CDK1 and BUB1 predicts poor prognosis of pancreatic ductal adenocarcinoma. Gene. 2019; 701:15–22. 10.1016/j.gene.2019.02.081 30898709

[32] Dong S, Huang F, Zhang H, Chen Q. Overexpression of BUB1B, CCNA2, CDC20, and CDK1 in tumor tissues predicts poor survival in pancreatic ductal adenocarcinoma. Biosci Rep. 2019; 39:BSR20182306. 10.1042/BSR20182306 30765611 PMC6390130

[33] Tian X, Wang N. Upregulation of ASPM, BUB1B and SPDL1 in tumor tissues predicts poor survival in patients with pancreatic ductal adenocarcinoma. Oncol Lett. 2020; 19:3307–15. 10.3892/ol.2020.11414 32218868 PMC7068710

[34] Fu X, Chen G, Cai ZD, Wang C, Liu ZZ, Lin ZY, Wu YD, Liang YX, Han ZD, Liu JC, Zhong WD. Overexpression of BUB1B contributes to progression of prostate cancer and predicts poor outcome in patients with prostate cancer. Onco Targets Ther. 2016; 9:2211–20. 10.2147/OTT.S101994 27143916 PMC4844448

[35] Stahl D, Braun M, Gentles AJ, Lingohr P, Walter A, Kristiansen G, Gütgemann I. Low BUB1 expression is an adverse prognostic marker in gastric adenocarcinoma. Oncotarget. 2017; 8:76329–39. 10.18632/oncotarget.19357 29100315 PMC5652709

[36] Zhuang L, Yang Z, Meng Z. Upregulation of BUB1B, CCNB1, CDC7, CDC20, and MCM3 in Tumor Tissues Predicted Worse Overall Survival and Disease-Free Survival in Hepatocellular Carcinoma Patients. Biomed Res Int. 2018; 2018:7897346. 10.1155/2018/7897346 30363964 PMC6186344

[37] Gayyed MF, El-Maqsoud NM, Tawfiek ER, El Gelany SA, Rahman MF. A comprehensive analysis of CDC20 overexpression in common malignant tumors from multiple organs: its correlation with tumor grade and stage. Tumour Biol. 2016; 37:749–62. 10.1007/s13277-015-3808-1 26245990

[38] Silva PMA, Delgado ML, Ribeiro N, Florindo C, Tavares ÁA, Ribeiro D, Lopes C, do Amaral B, Bousbaa H, Monteiro LS. Spindly and Bub3 expression in oral cancer: Prognostic and therapeutic implications. Oral Dis. 2019; 25:1291–301. 10.1111/odi.13089 30866167

[39] Ersvær E, Kildal W, Vlatkovic L, Cyll K, Pradhan M, Kleppe A, Hveem TS, Askautrud HA, Novelli M, Wæhre H, Liestøl K, Danielsen HE. Prognostic value of mitotic checkpoint protein BUB3, cyclin B1, and pituitary tumor-transforming 1 expression in prostate cancer. Mod Pathol. 2020; 33:905–15. 10.1038/s41379-019-0418-2 31801961 PMC7190565

[40] Haruki N, Saito H, Harano T, Nomoto S, Takahashi T, Osada H, Fujii Y, Takahashi T. Molecular analysis of the mitotic checkpoint genes BUB1, BUBR1 and BUB3 in human lung cancers. Cancer Lett. 2001; 162:201–05. 10.1016/s0304-3835(00)00675-3 11146226

[41] Morais da Silva S, Moutinho-Santos T, Sunkel CE. A tumor suppressor role of the Bub3 spindle checkpoint protein after apoptosis inhibition. J Cell Biol. 2013; 201:385–93. 10.1083/jcb.201210018 23609535 PMC3639401

[42] Rhodes DR, Yu J, Shanker K, Deshpande N, Varambally R, Ghosh D, Barrette T, Pandey A, Chinnaiyan AM. ONCOMINE: a cancer microarray database and integrated data-mining platform. Neoplasia. 2004; 6:1–6. 10.1016/s1476-5586(04)80047-2 15068665 PMC1635162

[43] Tang Z, Li C, Kang B, Gao G, Li C, Zhang Z. GEPIA: a web server for cancer and normal gene expression profiling and interactive analyses. Nucleic Acids Res. 2017; 45:W98–102. 10.1093/nar/gkx247 28407145 PMC5570223

[44] Ghandi M, Huang FW, Jané-Valbuena J, Kryukov GV, Lo CC, McDonald ER 3rd, Barretina J, Gelfand ET, Bielski CM, Li H, Hu K, Andreev-Drakhlin AY, Kim J, et al. Next-generation characterization of the Cancer Cell Line Encyclopedia. Nature. 2019; 569:503–08. 10.1038/s41586-019-1186-3 31068700 PMC6697103

[45] Sun CC, Li SJ, Hu W, Zhang J, Zhou Q, Liu C, Li LL, Songyang YY, Zhang F, Chen ZL, Li G, Bi ZY, Bi YY, et al. Comprehensive Analysis of the Expression and Prognosis for E2Fs in Human Breast Cancer. Mol Ther. 2019; 27:1153–65. 10.1016/j.ymthe.2019.03.019 31010740 PMC6554685

[46] Huang DW, Sherman BT, Tan Q, Collins JR, Alvord WG, Roayaei J, Stephens R, Baseler MW, Lane HC, Lempicki RA. The DAVID Gene Functional Classification Tool: a novel biological module-centric algorithm to functionally analyze large gene lists. Genome Biol. 2007; 8:R183. 10.1186/gb-2007-8-9-r183 17784955 PMC2375021

[47] Kanehisa M. The KEGG database. Novartis Found Symp. 2002; 247:91–101. 10.1002/0470857897.ch8 12539951

[48] Ashburner M, Ball CA, Blake JA, Botstein D, Butler H, Cherry JM, Davis AP, Dolinski K, Dwight SS, Eppig JT, Harris MA, Hill DP, Issel-Tarver L, et al. Gene ontology: tool for the unification of biology. The Gene Ontology Consortium. Nat Genet. 2000; 25:25–29. 10.1038/75556 10802651 PMC3037419

